# Case report and literature review: Primary leiomyosarcoma of the penis

**DOI:** 10.3389/fsurg.2022.1068935

**Published:** 2023-01-09

**Authors:** Yichang Hao, Li Xia, Min Lu, Chenhong Liu, Fan Zhang, Ye Yan, Yi Huang, Shudong Zhang

**Affiliations:** ^1^ Department of Urology, Peking University Third Hospital, Beijing, China; ^2^ Department of Pathology, Peking University Third Hospital, Beijing, China

**Keywords:** penis, leiomyosarcoma (LMS), case report, malignant mesenchymal tumors, urogenital neoplasms

## Abstract

**Background:**

Leiomyosarcoma (LMS) is a malignant spindle-cell mesenchymal tumor originating from the smooth muscle cells, which mostly affects soft tissues and abdominopelvic organs over extremities. Primary LMS of the penis is a relatively uncommon mesenchymal tissue disease and a poorly understood condition.

**Case Report:**

A 69-year-old man presented with a growing, painless mass protruding from the penis. The irregularly lobulated lump was roughly 3 cm × 2.5 cm, with a smooth surface, tough texture, distinct boundary, and no tenderness. It was determined to be a penile tumor during the preoperative radiological evaluation. The patient underwent resection of the penile mass, followed by extended resection in the second operation. The diagnosis of LMS was verified by pathological examination. During a 20-month follow-up, the patient made a smooth recovery and remained disease-free.

**Conclusion:**

An immunohistochemical examination is essential for rendering this rare diagnosis. Radical excision of tumor lesions with negative cut margins is guaranteed to be the best treatment for primary penile LMS. Close follow-up should be provided due to the high rate of local recurrence.

## Introduction

Malignant tumors of the penis are relatively rare, with an incidence of about 1 per 100,000 in developed countries such as North America and Europe; however, the incidence in less economically developed regions such as Asia, Africa, and South America is slightly higher than that in the aforementioned developed regions. The majority of penile malignant tumors (95%) are squamous cell carcinoma, while adenocarcinoma, malignant melanoma, and sarcoma are sporadic (1). Malignant mesenchymal tissue tumors (including Kaposi’s sarcoma, smooth muscle sarcoma, rhabdomyosarcoma, and malignant fibrous histiocytoma) account for less than 5%, among which primary leiomyosarcoma (LMS) of the penis is incredibly uncommon, mostly affecting middle-aged and older males (2). In 1969, Pratt and Ross (3) first classified LMS of the penis into deep and superficial types according to the tumor site. Since deep LMS has early metastasis and poor prognosis, early diagnosis and correct identification of the type are crucial.

A 69-year-old patient with primary penile LMS was admitted to our hospital in March 2021. This article, which is based on the CARE Guideline (4) and includes a reporting checklist in the supplementary material, analyzes the case data of this patient and reviews the pertinent literature to discuss the clinical manifestations, diagnosis, and treatment of primary penile LMS in order to better understand and diagnose it.

## Case presentation

A 69-year-old male patient was admitted to the hospital with a 3-year painless mass in the penis as his main complaint. Three years ago, the patient had a soy bean-sized, painless mass in the middle shaft of the penis with no obvious cause. No ulceration or effusion, urinary frequency, urgency, or urinary pain was present nor was there a fever or any other discomfort. The lump rapidly enlarged to a diameter of 3 cm 6 months ago, without any swelling, heat, pain, or any other discomfort. The patient had a history of hypertension and type 2 diabetes mellitus, both of which responded effectively to oral treatment.

Physical examination revealed an irregular lobulated mass measuring about 3 cm × 2.5 cm in the middle shaft part of the penis on the ventral side, with a smooth surface, tough texture, clear border, a moderate range of motion, and no tenderness. There was no visible skin ulceration on the surface, no redness or swelling of urethral orifice, and no aberrant secretion. The epididymis and bilateral testis were both normal, and there were no swollen lymph nodes in the inguinal region. The skin color of the scrotum was also normal.

**Figure 1:**
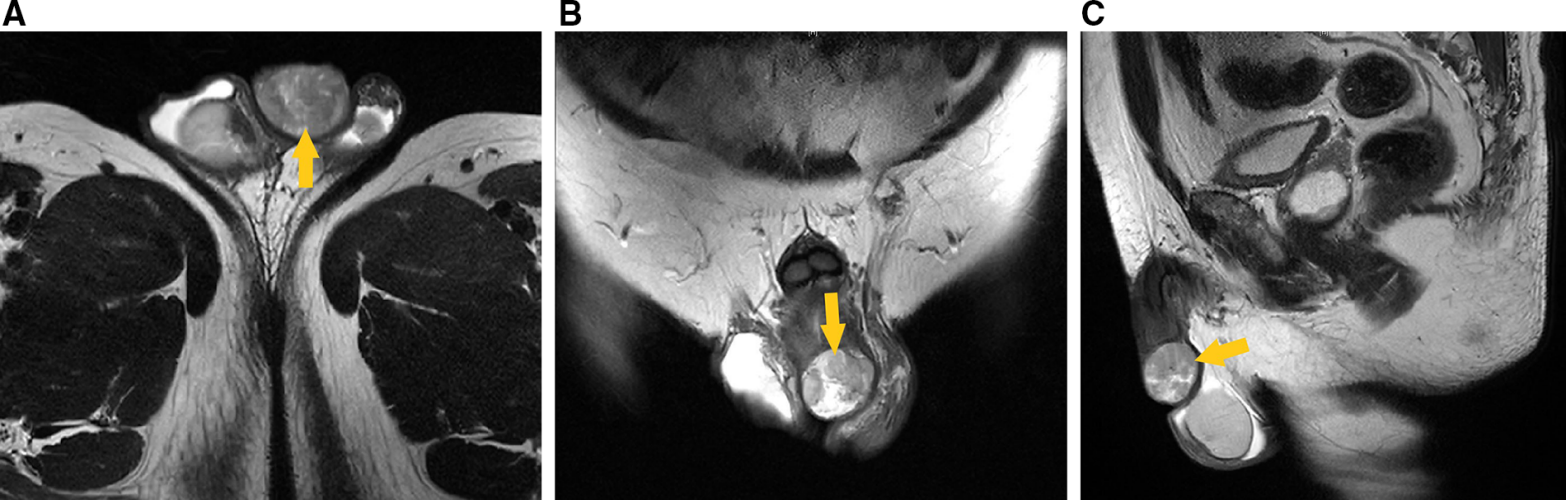
Preoperative pelvic MRI (male genital system) showed nodular mixed signal shadow in the distal penis (arrow head), with a size of about 31 mm × 27 mm × 22 mm, and a high signal on DWI. (**A**) Transverse section, T2-weighted imaging. (**B**) Coronal section, T2-weighted imaging. (**C**) Median sagittal section, T2-weighted imaging.

Auxiliary examination revealed that the usual tests for blood, urine, stool, liver and kidney function, electrolytes, myocardial enzymes, coagulation function, and prostate-specific antigen were all normal. Preoperative pelvic magnetic resonance imaging (MRI) (male genital system) showed a nodular mixed signal shadow in the distal penis, with a size of about 31 mm × 27 mm × 22 mm, and a high signal on diffusion-weighted imaging (DWI). No noticeable large lymph nodes were seen in the pelvis (Figure 1). Preoperative ultrasonography showed that no enlarged lymph nodes were detected in the bilateral inguinal region.

Under intralesional anesthetic, the patient had the penile lump removed. The mass was in the lower fascia layer (Eberth fascia), totally excised, and there was no visible adhesion between it and the surrounding tissues.

**Figure 2:**
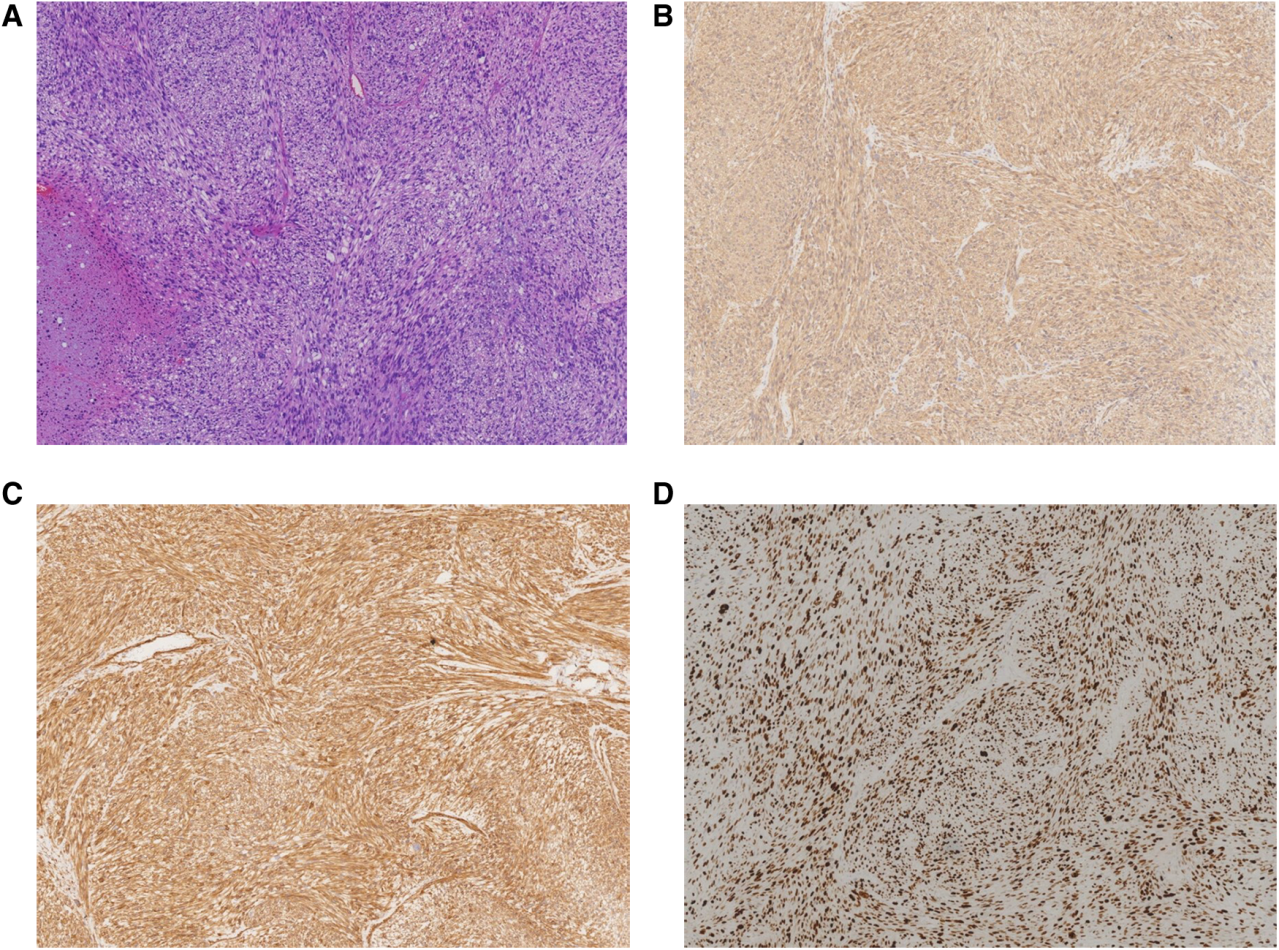
Pathological examination showing that the mass was composed of atypical spindle-shaped cells tumor with focal necrosis. (**A**) Hematoxylin and eosin (HE) staining with high mitoses and atypical mitoses (×40). (**B**) Immunohistochemistry staining of SMA (×40). (**C**) Immunohistochemistry staining of Caldesmon (×40). (**D**) Immunohistochemistry staining of Ki-67 (×40).

Gross examination revealed a grey-yellow necrotic tumor, measuring 3 cm × 3 cm × 2.5 cm. Microscopic examination showed severe atypia and high mitoses. Immunohistochemical results showed that S-100 (−), CD34 (−), Ki-67 (about 70% +), P53 (consistent with wild type), smooth muscle actin (SMA) (+), Desmin (+), and Caldesmon (+). Results from immunohistochemistry and morphology were consistent with LMS (Figure 2).

Three weeks later, the patient was readmitted and an extended resection was performed under endotracheal anesthesia based on sufficient dialog with the patient and his families. The skin was excised from the surgical site within 3 cm, and subcutaneous tissue was dissected all the way to Buck’s fascia. Postoperative pathology suggested no residual tumor cells.

There was no local discomfort after surgery and slight scarring at the wound; postoperative erectile function was basically the same as before. No local tumor recurrence, inguinal lymph node enlargement, and metastasis were noticed over the 20-month follow-up.

## Discussion

LMS is one of the most common subtypes of malignant mesenchymal tissue tumors, accounting for approximately 10%–20% of soft tissue sarcomas, which often affects the abdomen, retroperitoneum, large vessel wall, and uterus (5), and rarely involves the penis. Primary sarcomas of the penis also include Kaposi’s sarcoma, epithelioid hemangioendothelioma, hemangiosarcoma, and rhabdomyosarcoma. Since Levi’s initial report in 1930, 61 cases of primary LMS of the penis have been reported in English (Table 1), ranging in age from 6 to 84 years, with cases most frequently occurring in people in their forties and fifties (6–9). Penile LMS often originates from the following structures: (1) the dermal layer of the erector spinae; (2) the superficial fascial muscular layer of the penis; (3) the muscular layer of the superficial vessels outside the tunica albuginea; and (4) the muscular layer of the deep vascular complex that make up the corpus cavernosum and corpus spongiosum. With the tunica albuginea acting as the boundary, it can be divided into deep LMS and superficial LMS. Superficial LMS mostly appears on the surface of the distal penis or glans and is characterized by painless nodules, sluggish development, and a few deep infiltrations. Deep LMS, however, can involve the smooth muscle of the corpus cavernosum and invade the urethra and other surrounding structures (10).

**Table 1:** Reported cases of leiomyosarcoma of the penis.

Case	Author	Age (years)	Tumor size (cm)	Anatomic location	Treatment	Course and follow-up
1	Levi (1930) (11)	38	NA (small nodule)	Superficial on dorsum of distal shaft	Small nodule since childhood. Local excision at 36	No follow-up
2	Kreibig (1931) (12)	46	NA (small nodule)	Superficial on dorsum of distal shaft	At ages 12 and 39, small nodule removed from prepuce; histology unknown. At 46, radical amputation after failed radiation	No follow-up
3	Meller (1932) (13)	64	NA	Superficial, proximal to coronal sulcus	Radiotherapy and eventual amputation	No follow-up
4	Ashley and Edwards (1957) (14)	49	NA (>2)	Superficial, proximal to coronal sulcus	Local excision followed by recurrence after 18 years	Well 18 months after second operation
5	Fagundes et al. (1962) (15)	52	NA	Deep, adherent to urethra at root of penis	Local excision with three recurrences over 5 years. Radiotherapy and eventual amputation	Well 10 months after amputation
6	Izdebski and Wiercinski (1962) (16)	31	NA	Deep, adherent to corpus cavernosum	Refused treatment	Dead 3 years later with widespread metastases
7	Pack et al. (1963) (17)	83	4	Deep, root of penis	Radiotherapy and eventual amputation	Dead 2 years later with lung metastases
8	Chaudhuri and Balasubrahmanyan (1966) (18)	40	6	Superficial on left side of prepuce	Radical amputation	Alive, well 3 years later
9	Bakken et al. (1968) (19)	60	19	Deep, bulbous portion of corpus spongiosum	Radical amputation	Dead 6 months later with widespread metastases
10	Hutcheson et al. (1969) (20)	63	NA (>4.5)	Superficial, dorsal aspect of midshaft of penis	Local excision with recurrences at 3 and 8 years. Radiotherapy and eventual amputation	No recurrence after radical excision
11	Pratt and Ross (1969) (3)	38	3	Deep, root of penis	Radical amputation	Alive, well 2 years after operation
12	Dehner and Smith (1970) (1)	67	NA	Superficial on dorsal shaft of distal penis	Local excision	No follow-up
13	Dehner and Smith (1970) (1)	45	3	Superficial on dorsum of shaft	Local excision	Well 1 year later
14	Greenwood et al. (1972) (21)	64	NA	Superficial near frenulum	Radiotherapy. Eventual partial amputation 2 years later	Alive, well 1 year after operation
15	Glucker et al. (1972) (22)	6	NA	Superficial, dorsum of glans	Local excision	Alive, free from recurrence at 1 year
16	Nkposong and Osunkoya (1972) (23)	52	NA	Deep in right corpus cavernosum	Local excision	Dead 3 months after operation
17	Gupta et al. (1973) (24)	60	10.5	Deep in distal part of shaft and glans	Radical amputation	Dead 5 months later with widespread metastases
18	Prabhakar et al. (1975) (25)	54		Deep, anterior portion of shaft	Penectomy	Dead 1 month after operation with lung metastases
19	Hamal (1975) (26)	84	NA	Glans penis at the coronal sulcus	Partial amputation	Alive, free from recurrence for 6 months
20	Blath and Manley (1975) (27)	44	5 (multinodular)	Prepuce	Wide circumcision	Well 1 year later and free from recurrence
21	Armijo et al. (1978) (28)	70	NA	Glans and prepuce	Local excision, followed by another local excision	No recurrence after 8 months
22	Elem and Nieslanik (1979) (29)	60	NA	Deep, corpus spongiosum	Partial amputation	Dead 1 month after operation with lung metastases
23	Weinberger et al. (1982) (30)	47	1	Superficial, proximal to coronal sulcus	Local excision	Well 9 months after surgery
24	Jain et al. (1982) (31)	55	5	Superficial involving the glans of penis	Total amputation	Well 18 months after surgery
25	McDonald et al. (1983) (32)	62	8	Deep in right corpus cavernosum	Chemotherapy followed by emasculation and radiotherapy	Dead 7 months after initial diagnosis
26	Isa et al. (1984) (33)	39	NA (small nodule)	Superficial on dorsal shaft of penis	Local excision, then partial penectomy. Right groin dissection with radiotherapy 18 years later	Alive, well 1 year following groin dissection
27	Smart (1984) (34)	61	NA	Deep, root of penis	Radical penectomy and radiotherapy	Well at 6 months
28	Valadez and Waters (1986) (35)/Kathuria et al. (1986) (36)/Fetsch et al (2004) (37)	45	2	Superficial on prepuce	Circumcision	Well at 18 months
29	Koizumi, et al. (1987) (38)	53	NA	Glans	Partial penectomy	Well at 12 months
30	Pow-Sang and Orihuela (1994) (39)	44	NA	Superficial on prepuce	Wide circumcision	Well at 28 months
31	Dobos et al. (2001) (40)	38	2.5, 0.4, and 0.3	Glans and shaft	Radical amputation	Alive with widespread metastases, 6 months after surgery
32	Katsikas et al. (2002) (8)	78	8, 8, and 14	Penile root, midshaft	Radical penectomy	No recurrence after 2 years
33	Dominici et al. (2004) (10)	53	2 × 1	Superficial on prepuce	Circumcision. Partial penectomy at 4 year recurrence	Well 12 months after second operation
34	Fetsch et al. (2004) (37)	43	2	Circumcision scar	Local excision	NA
35	Fetsch et al. (2004) (37)	45	2.4	Dorsolateral midshaft	Local excision	NA
36	Fetsch et al. (2004) (37)	49	1.5	Circumcision scar and distal shaft	Local excision	Recurrence after 1 year → local excision → recurrence after 3 years → partial amputation → no recurrence after 5 years 8 months
37	Fetsch et al. (2004) (37)	53	0.5	Base of shaft at junction with abdominal wall	Local excision	No recurrence after 5 years 2 months
38	Fetsch et al. (2004) (37)	53	0.9	Lateral shaft, near base	Local excision	No recurrence after 11 years
39	Fetsch et al. (2004) (37)	59	1.2	Prepuce and distal shaft	Local excision	NA
40	Fetsch et al. (2004) (37)	61	2	Shaft	Local excision	No recurrence after 13 years 11 months
41	Fetsch et al. (2004) (37)	62	0.7	Circumcision scar	Local excision	No recurrence after 16 years 1 month
42	Fetsch et al. (2004) (37)	43	Multiple pieces to 2	Periurethral (shaft)	Local excision	No recurrence after 18 years 7 months
43	Fetsch et al. (2004) (37)	47	1.5	Shaft	Local excision, local excision, wide local excision	Recurrence after 2 years 4 months → local excision → recurrence after 3 months → wide local excision → no recurrence after 10 years 4 months
44	Fetsch et al. (2004) (37)	48	1.5	Penis, NOS	Local excision	NA
45	Fetsch et al. (2004) (37)	58	6	Penile root	Local excision	NA
46	Fetsch et al. (2004) (37)	NA	NA	Shaft	Local excision 5 and lymph node dissection	Local recurrence 4, followed 10 months later by a metastasis to left arm, then lost to follow-up
47	Mendis et al. (2005) (41)	51	NA	Glans	Local excision	No recurrence after 5 months
48	Nanri et al. (2006) (42)	27	10 × 7	Deep in penile root	Total penectomy and chemotherapy	died from disseminated disease 14 months after surgery
49	Cibull et al. (2008) (6)	68	1.5	Glans	Partial penectomy	No recurrence after 13 months
50	Sundersingh et al. (2009) (43)	56	3.5 × 3 × 3	Deep, glans and distal shaft	Total penectomy	No recurrence after 6 months
51	Lacarrière et al. (2011) (44)	64	NA (bilateral masses)	Deep, penile root	Local excision, total penectomy, and chemotherapy	Lung metastases after 2 months operation
52	Brisciani et al. (2012) (45)	63	1.3 nodule	Distal shaft	Local excision	No recurrence after 3 months
53	Cigna et al. (2013) (2)	62	2.2 × 1.5	Dorsum of glans	Local excision	No recurrence after 1 year
54	D'Cruze et al. (2014) (46)	59	4	Glans	Partial penectomy	No recurrence after 11 months
55	Romero Gonzalez et al. (2015) (47)	39	1	Distal shaft	Local excision	No recurrence after 3 years 6 months
56	Khobragade et al. (2015) (9)	26	4.7 × 3.7 × 5.4	Penile root	Local excision	No recurrence after 2 years
57	Khobragade et al. (2015) (9)	38	3 × 4 (multinodular)	Glans, proximal shaft	Total penectomy	No recurrence after 9 months
58	Rabinovich (2018) (48)	39	2	Superficial, prepuce	Wide circumcision	No recurrence after 9 months
59	Ajmal et al. (2022) (49)	70	3 × 2.2 × 1.5	Prepuce	circumcision	NA
60	da Costa Junior et al. (2022) (50)	54	2.0 × 1.0	Glans	Local excision (the surgical margin had neoplasm)	No recurrence after 5 months
61	Goyal et al. (2022) (51)	70	6.5 × 5.5	Glans, distal shaft	Partial penectomy	NA
62	Current report (2022)	69	3 × 2.5	Superficial, shaft	Local excision	No recurrence after 17 months

NA, not available; NOS, not otherwise specified.

LMS of the penis can present with a variety of clinical manifestations, such as painless nodules or masses, localized pain and swelling, superficial skin ulceration, hematuria, urethral obstruction, and enlargement of inguinal lymph node. In comparison to superficial LMS, deep LMS located below the deep fascia of the penis are more likely to manifest with hematuria and urethral obstruction (10). The most frequent location of tumor was the shaft, followed by the prepuce, whereas the coronal sulcus, the frenulum, and circumcision scars were additional uncommon locations (2).

Due to the rarity of penile LMS and the lack of distinct clinical symptoms, tumor excision and biopsy are typically required for diagnosis. A careful inspection of the lesion and palpation of the inguinal lymph nodes are required if a clinical suspicion of soft tissue sarcoma is strong. It is preferable to use MRI to demonstrate the depth of tumor invasion and evaluate both inguinal and pelvic lymph nodes. Deep LMS is prone to blood metastases, with the lung and liver being the most common sites of metastasis; therefore, a simultaneous CT scan of the chest and abdomen is recommended for high-risk tumors.

The pathologic diagnosis often includes a pretreatment biopsy and further pathologic evaluation after the tumor has been surgically removed to differentiate it from other sarcomas (52). It is important to note that fine needle aspiration frequently yields inadequate tissue to make a diagnosis. LMS consists of cells with a smooth muscle spectrum; both superficial and deep LMS have the same characteristic histologic features: spindle-shaped cells, with eosinophilic cytoplasm, long rod-shaped and darkly stained nuclei (53). The mitotic rate and other mitotic variables could predict the tendency of tumor invasion to adjacent structures or metastasis (8). In immunohistochemistry, SMA, Desmin, and Caldesmon are typically positive, but none of these markers are specific for smooth muscle differentiation (53). Cytokeratin and S100 were negative and could be differentiated from epithelial tissue (39). Negative CD34 could be identified from Kaposi’s sarcoma (46). Immunopositive results for P16 and P53 with high Ki-67 proliferation index are highly sensitive and specific for the distinction of LMS and leiomyoma (52).

The principle of treatment for primary penile LMS is currently considered to be radical resection of the tumor lesion (R0 surgery) with maximum local organs preservation. Whether local lesion excision, partial penectomy, or total penectomy is performed depends on tumor type, size, and presence of metastasis. Tumor size is one of the best predictors of outcome for primary LMS of penis, when stratified as follows: ≤2 vs. >2 cm and ≤5 vs. >5 cm (37). First, local lesion and extensive resection is the best choice for superficial LMS and ≤2 cm in diameter, and the prognosis is better because distant metastasis of superficial LMS is rare. Due to the risk of recurrence, it is important to ensure a safe margin. The most critical factor for recurrence-free survival is the microscope-negative tumor margin. The majority of studies recommend a margin of at least 1 cm, while some have found that a margin of 2–5 cm is associated with a decreased rate of recurrence after resection (54). For subcutaneous LMS of the skin, it is recommended and desirable for complete excision of the subcutaneous tissue with at least 2–3 cm of the skin margin and subcutaneous tissue (55). A second surgery was performed in our patient that included a deep subcutaneous tissue excision that reached the penile fascia (Buck’s fascia) and an expanded excision of the skin that was removed within 3 cm of the surgical site margin. However, partial penile resection or radical excision is typically the mainstay of treatment for profound LMS (9). When distant metastasis has occurred, the aim of treatment includes symptom relief, tumor volume reduction, and prolonging survival. Because of rare local lymph node metastasis in LMS and the distant metastasis is often present when the peripheral lymph nodes are involved, regional lymph node dissection is not advised in the absence of clearly clinical or imaging evidence of lymph node metastasis (52).

Adjuvant radiation treatment (RT) and chemotherapy may help in the treatment of LMS in order to preserve organ function and reduce local recurrence, although with a limited impact on survival rates (52). In a retrospective study of 14 patients with primary penile LMS in 1994 (39), local tumor recurrence was found in all patients treated with chemotherapy or radiotherapy only, and distant metastasis was found in 2 of them. Hensley et al. (56) reported that gemcitabine combined with docetaxel chemotherapy was significantly effective as first- and second-line treatment for primary penile LMS. The effectiveness of adjuvant radiation and chemotherapy in treating primary penile LMS has not been verified because of the small number of cases.

Tumor size, tumor depth, and histologic grade are the main factors affecting the risk and prognosis of primary penile LMS, as with other soft tissue sarcomas. The American Joint Committee on Cancer (AJCC) grading system has a grading system that takes into account characteristics such as tumor size, lymph node involvement, and distant metastasis; however, no research has determined if penile LMS falls within this system. In the TNM grading system, T stage is divided into T1 and T2 stages with the maximum diameter of 5 cm, but the majority of penile LMS is less than 5 cm. Thus, a lower cut-off value could be more applicable. In addition, for penile LMS, T stage should distinguish the superficial type from the deep type according to the depth of invasion, rather than just by the size of tumor. Moreover, poor differentiation (grade 3 or 4) results in upstaging to stages II or III irrespective of the tumor size (55). The Fédération Nationale des Centres de Lutte Contre Le Cancer (FNCLCC) grading system is the most widely used in the histological grading of soft tissue sarcomas. Based on the degree of differentiation, mitosis, and tumor necrosis, the FNCLCC grading can be divided into X, 1, 2, and 3 grades. The higher the grade is, the worse the prognosis will be (57). Eventually, superficial penile LMS has a better prognosis than deep LMS, especially for primary LMS with infiltration depth ≤2 cm and size ≤5 cm and being treated by extensive local excision with negative incisional margins. However, larger and deep LMS, especially located at the root of the penis, usually have a poor prognosis (33).

Local recurrence may occur after the surgery of primary penile LMS, and tumor cells often become poorly differentiated after recurrence. For superficial and deep lesions, the recurrence rates are 23% and 29%, respectively. However, the risk rate of distant metastasis was higher for deep-type LMS (50%) compared to 8% for superficial LMS. In addition, the risk of metastasis increased with tumor size, with a 29% and 50% chance of metastasis for tumors with a diameter of 5 cm and greater. Local lymph node metastasis is uncommon and occurs mostly in the advanced stages of the disease, when distant metastases are often already present and the prognosis is poor (37).

Follow-up guideline for primary penile LMS are little standardized and adapted from the soft tissue sarcomas in general (55). A complete examination, especially the operation site and inguinal lymph nodes, should be carried out every 3 months for 3 years after resection, every 6 months for the following 2 years, and then annually for up to 10 years. In high-risk cases (>5 cm tumor size, deep LMS, local relapse, high-grade LMS), chest CT should be performed every 3–6 months together with MRI of the primary tumor site and sonography of regional lymph nodes and abdomen.

Additionally, the patient underwent surgery twice. As an improvement measure, it is recommended to be alert to the possibility of malignant mass, particularly given its rapid growth during the last period of the present case, and intraoperative frozen section analysis is necessary. In addition, after local excision, expanded resection of the margins and base of the lesion for biopsy is advised.

## Conclusion

In summary, though primary LMS of penis is very rare, it is not difficult to diagnose when pathology is included. Patients with deep lesions are likely to experience distant metastases at an early stage, which often has a bad prognosis. The best therapy for primary penile LMS to date is assured to be radical removal of malignant lesions with negative cut margins, and close monitoring should be administered.

## Data Availability

The original contributions presented in the study are included in the article/Supplementary Material, further inquiries can be directed to the corresponding authors.
